# Prognosis after percutaneous coronary intervention in patients with psoriasis: a cohort study using Danish nationwide registries

**DOI:** 10.1186/1471-2261-12-79

**Published:** 2012-09-24

**Authors:** Ole Ahlehoff, Jesper Lindhardsen, Gunnar H Gislason, Jonas B Olesen, Mette Charlot, Lone Skov, Christian Torp-Pedersen, Peter R Hansen

**Affiliations:** 1 Department of Cardiology, Copenhagen University Hospital, Gentofte, Hellerup DK-2900, Denmark; 2 Department of Cardiology, Copenhagen University Hospital, Roskilde, Roskilde DK-4000, Denmark; 3 Department of Dermatology, Copenhagen University Hospital, Gentofte, Hellerup DK-2900, Denmark

**Keywords:** Coronary revascularization, Cardiovascular disease, Inflammation, Psoriasis, Prognosis

## Abstract

**Background:**

Psoriasis is an inflammatory disease associated with increased risk of coronary artery disease. However, the potential impact of psoriasis on the prognosis following percutaneous coronary revascularization (PCI) is unknown.

**Methods:**

The study comprised the entire Danish population undergoing first-time PCI in the period 2002–09. Cox regression models, controlling for age, gender, socioeconomic status, pharmacological treatment, and comorbidity were used to assess the risk of 1) all-cause mortality and 2) a composite endpoint of death, myocardial infarction, and stroke.

**Results:**

A total of 53,141 patients with first-time PCI in the study period were identified. Of these, 1074 had mild psoriasis and 315 had severe psoriasis. Patients with severe psoriasis, but not those with mild disease had increased risk of both endpoints compared to patients without psoriasis. The incidence rates for all-cause mortality were 30.5 (CI 29.7-31.3), 29.9 (CI 24.7-36.1), and 47.2 (CI 35.0-63.6) per 1000 patient years for patients without psoriasis, with mild psoriasis, and with severe psoriasis, respectively. Hazard ratios were 1.10 (CI 0.91-1.33) and 1.67 (CI 1.24-2.26) for mild and severe psoriasis, respectively. Patients with severe psoriasis were less likely to receive secondary prevention pharmacotherapy with betablockers, statins and platelet inhibitors.

**Conclusion:**

This first study of the prognosis following PCI in patients with psoriasis demonstrated an increased risk of all-cause mortality and of a composite of death, myocardial infarction and stroke, respectively, in patients with severe psoriasis compared to patients without psoriasis. Further studies of this novel association are needed.

## Background

Psoriasis is a chronic inflammatory disease affecting the skin of 1-3% of the population [1]. Other manifestations of psoriasis include scalp psoriasis, nail disease, and sero‐negative arthritis known as psoriatic arthritis [1]. Atherosclerosis is also regarded as an inflammatory disease and the underlying mechanisms are very similar to those seen in psoriasis [2,3]. Furthermore, psoriasis is associated with conventional cardiovascular risk factors, including diabetes mellitus, hypertension, and dyslipidemia [4,5]. Like other inflammatory disorders including rheumatoid arthritis and systemic lupus erythromatosus, psoriasis confers independent risk of atherothrombotic disease, invasive coronary revascularization, and cardiovascular mortality [6-11]. In the Danish population, patients with psoriasis have been demonstrated to have an increased (1.4 to 2.3 fold) risk of coronary revascularization, including percutaneus coronary intervention (PCI) [9]. Recent results have indicated that patients with psoriasis face a poorer prognosis following myocardial infarction compared to patients without psoriasis [12]. Although PCI is widely used in the treatment of acute and chronic coronary artery disease there are no data available on the potential impact of psoriasis on post-PCI prognosis.

We therefore used the Danish nationwide registries to examine the psoriasis-related risks of death, myocardial infarction, stroke, and repeat revascularization in patients with and without psoriasis after first-time PCI in the time-period 2002–2009.

## Results

Baseline characteristics of the study population are presented in Table 1. A total of 53,141 patients, including 1074 with mild psoriasis and 315 with severe psoriasis, with first-time PCI in the study period were identified. Patients with severe psoriasis were slightly younger and more often women compared to patients without psoriasis. In approximately 55–60% the index PCIs were performed in patients with a previous myocardial infarction, including 26–29% primary PCI procedures, with no significant differences between patients with severe psoriasis and patients without psoriasis (Tables 1 and 2). In the majority (88.3%) of baseline previous myocardial infarctions the event date was less than 30 days from the index PCI. At baseline, patients with psoriasis received more non steroid anti-inflammatory drugs (NSAIDs) and more antihypertensive, cholesterol-lowering, glucose-lowering, and antidepressive medication, respectively, than patients without psoriasis.

**Table 1:** Baseline characteristics of the study population

	**No psoriasis**	**Mild psoriasis**	**Severe psoriasis**	**p–value**
	**n=51,752**	**n=1074**	**n=315**	
**Male (%)**	36,989 (71.5)	767 (71.4)	193 (61.3)	0.003
**Female (%)**	14,763 (28.5)	307 (28.6)	122 (38.7)	
**Age, years (SD)**	65.1 (11.6)	65.0 (10.7)	63.5 (10.9)	0.06
**Treatment (%)**				
Beta-blocker	20,851 (40.3)	434 (40.4)	132 (41.9)	0.60
ACE inhibitor/ARB	16,868 (32.6)	381 (35.5)	121 (38.4)	0.003
Statin	21,462 (41.5)	506 (47.1)	136 (43.2)	0.003
Platelet inhibitor	21,209 (41.0)	490 (45.6)	133 (42.2)	0.02
Loop diuretic	6663 (12.9)	139 (12.9)	48 (15.2)	0.33
Spironolactone	1733 (3.4)	38 (3.5)	11 (3.5)	0.74
Vitamin K antagonist	2505 (4.8)	48 (4.5)	11 (3.5)	0.23
Glucose lowering medication	5734 (11.1)	123 (11.5)	52 (16.5)	0.01
NSAID	3465 (6.7)	101 (9.4)	36 (11.4)	<0.001
Antidepressive medication	4569 (8.8)	125 (11.6)	42 (13.3)	<0.001
**Comorbidity (%)**				
Myocardial infarction	31,078 (60.1)	596 (55.5)	182 (60.0)	0.008
Cardiac dysrhythmia	4759 (9.2)	115 (10.7)	28 (3.2)	0.33
Diabetes with complications	2117 (4.1)	38 (3.5)	22 (7.0)	0.20
Cerebrovascular disease	1373 (2.7)	33 (3.1)	10 (3.2)	0.32
Pulmonary edema	187 (0.4)	5 (0.5)	2 (0.6)	0.33
Shock	593 (1.2)	6 (0.6)	8 (2.5)	0.62
Cancer	424 (0.8)	9 (0.8)	3 (1.0)	0.62
Acute renal failure	331 (0.6)	2 (0.2)	3 (1.0)	0.46
Chronic renal failure	592 (1.1)	8 (0.2)	4 (1.1)	0.51
Chronic renal failure	1904 (3.7)	50 (4.7)	15 (4.8)	0.06
pulmonary disease				

ACE: angiotensin-converting enzyme inhibitor; ARB: angiotensin 2 receptor blocker; NSAID: Non-steroid anti-inflammatory drug.

**Table 2:** Post PCI characteristics at 30, 180, and 360 days from index PCI

**30 days after PCI**	**No psoriasis**	**Mild psoriasis**	**Severe psoriasis**	**p–value**
	**n= 49,696**	**n=1027**	**n=309**	
**Male (%)**	35,632 (72.7)	737 (72.8)	192 (62.1)	0.007
**Female (%)**	14,064 (28.3)	290 (28.2)	117 (37.9)	
**Primary PCI (%)**	14,451 (29.1)	268 (26.1)	90 (29.1)	0.17
**Treatment (%)**				
Beta-blocker	38,916 (78.3)	772 (75.2)	230 (74.4)	0.005
ACE inhibitor/ARB	25,032 (50.4)	509 (49.6)	167 (54.1)	0.54
Statin	42,844 (86.2)	896 (86.3)	245 (79.3)	0.049
Platelet inhibitor	47,949 (96.5)	991 (96.5)	285 (92.2)	0.003
Dual anti-platelet inhibition	45,901 (92.4)	946 (92.1)	273 (88.4)	0.029
Loop diuretic	9812 (19.7)	201 (19.6)	71 (23.0)	0.34
Spironolactone	2852 (5.7)	53 (5.2)	23 (7.4)	0.67
Vitamin K antagonist	2904 (5.8)	58 (5.7)	18 (5.8)	0.85
Glucose-lowering medication	5586 (11.2)	112 (10.9)	52 (16.8)	0.039
NSAID	1349 (2.7)	40 (3.9)	34 (11.0)	0.002
**180 days after PCI**	**No psoriasis**	**Mild psoriasis**	**Severe psoriasis**	**p–value**
	**n= 46,126**	**n=955**	**n=279**	
Beta-blocker	36,431 (78.9)	727 (76.1)	212 (76.0)	0.05
ACE inhibitor/ARB	23,324 (50.6)	484 (50.7)	153 (54.8)	0.36
Statin	40,140 (87.0)	842 (88.2)	226 (81.0)	0.007
Platelet inhibitor	44,696 (96.9)	927 (97.1)	260 (93.2)	0.002
Dual anti-platelet inhibition	42,822 (92.8)	889 (93.1)	249 (89.3)	0.07
Loop diuretic	8968 (19.4)	188 (19.7)	62 (22.2)	0.50
**360 days after PCI**	**No psoriasis**	**Mild psoriasis**	**Severe psoriasis**	**p–value**
	**n= 42,188**	**n=860**	**n=257**	
Beta-blocker	33,507 (79.4)	665 (77.3)	195 (75.9)	0.124
ACE inhibitor/ARB	21,389 (50.7)	430 (50.0)	140 (54.5)	0.44
Statin	36,918 (87.5)	769 (89.4)	210 (81.7)	0.005
Platelet inhibitor	40,985 (97.2)	840 (97.7)	239 (93.0)	<0.001
Dual anti-platelet inhibition	39,318 (93.2)	811 (94.3)	228 (88.7)	228 (88.7)
Loop diuretic	8055 (19.1)	161 (18.7)	52 (20.3)	0.86

PCI: percutaneous coronary intervention; ACE: angiotensin-converting enzyme inhibitor; ARB: angiotensin 2 receptor blocker; NSAID: Non-steroid anti inflammatory drug.

Patients who survived to enter the sensitivity analyses with follow-up start at day 30 after the index PCI had a similar gender distribution and the prevalence of psoriasis in this analysis was similar (2.7%). There were also no differences in the proportion of (index) primary PCI procedures between the groups in this population. Patients with severe psoriasis generally received significantly less secondary prevention medication including beta-blockers, statins, and platelet inhibitors. Furthermore, as in the primary analysis, patients with psoriasis were more likely to receive NSAIDs, antidepressive medication, and glucose-lowering drugs. An overview of post-PCI treatment is presented in Table 2.

### Post PCI prognosis

Psoriasis was associated with an increased risk of both study endpoints (p-value for trend 0.004 and 0.007 for all cause mortality and the composite endpoint, respectively). In analyses stratified for psoriasis severity patients with severe psoriasis, but not those with mild disease had significantly increased prevalence of both study endpoints compared to patients without psoriasis. The incidence rates (IRs) and 95% confidence intervals (CIs) for all-cause mortality were 30.5 (CI 29.7-31.3), 29.9 (CI 24.7-36.1), and 47.2 (CI 35.0-63.6) for patients without psoriasis, with mild psoriasis, and with severe psoriasis, respectively. The corresponding IRs for the composite endpoint of death, myocardial infarction, and stroke were 38.9 (CI 38.1.8-39.9), 37.3 (CI 31.4-44.4), and 55.4 (CI 41.8-73.5). In the primary analysis with start of follow-up at the time of the index PCI and adjustment for age and sex, the hazard ratios (HRs) for all cause mortality were 1.10 (CI 0.91-1.33) and 1.83 (CI 1.36-2.47) for mild and severe psoriasis, respectively (Table 3). For the composite endpoint the corresponding age and sex adjusted HRs were 1.07 (CI 0.90-1.27) for mild psoriasis and 1.65 (CI 1.24-2.19) for severe psoriasis. The sensitivity analysis with start of follow-up 30 days from index PCI and inclusion of post-PCI medication in the Cox regression model gave similar results. The age- and sex-adjusted estimates for all cause mortality were HR 1.11 (CI 0.89-1.37) and HR 2.06 (CI 1.50-2.84), for mild and severe psoriasis, respectively. For the composite endpoint the corresponding age and sex adjusted HRs were 1.08 (CI 0.88-1.34) for mild psoriasis and 1.90 (CI 1.38-2.62) for severe psoriasis (Table 3).

**Table 3:** Number of events, incidence rates, adjusted HRs, and 95% CI

	**Number of events**	**Incidence rate**	**95% CI**	**Hazard ratio**	**95% CI**
**All cause mortality**					
***Follow-up start at index PCI***					
Mild psoriasis	107	29.9	24.7-36.1	1.10	0.91-1.33
Severe psoriasis	43	47.2	35.0-63.6	1.67	1.24-2.26
***Follow-up start at day 30***					
Mild psoriasis	85	24.3	19.7-30.1	1.13	0.91-1.40
Severe psoriasis	38	42.8	31.1-58.8	1.78	1.30-2.46
**Composite endpoint**					
***Follow-up start at index PCI***					
Mild psoriasis	128	37.3	31.4-44.4	1.07	0.90-1.28
Severe psoriasis	48	55.4	41.8-73.5	1.54	1.16-2.05
***Follow-up start at day 30***					
Mild psoriasis	90	26.9	21.9-33.1	1.10	0.90-1.36
Severe psoriasis	38	45.1	45.1 32.8-61.1	1.66	1.21-2.28

HR: hazard ratio; CI: confidence interval; PCI: percutaneous coronary intervention.

### Repeat revascularization

There was a nonsignificant trend (p = 0.23) for increased repeat revascularization rates in patients with psoriasis compared to patients without psoriasis. IRs were 66.9 (CI 65.6-68.3), 71.3 (CI 62.2-81.6), and 75.0 (CI 57.7-97.5), for patients without psoriasis, with mild psoriasis, and with severe psoriasis, respectively. The corresponding adjusted HRs were 1.05 (CI 0.89-1.22) and 1.14 (CI 0.85-1.52) for mild and severe psoriasis, respectively.

## Discussion

This nationwide study of the impact of psoriasis on prognosis following PCI demonstrated an increased risk of all-cause mortality and of a composite of death, myocardial infarction, and stroke, respectively, in patients with severe disease compared to subjects without psoriasis. The post-PCI prognosis in patients with mild psoriasis was comparable to that of patients without psoriasis. Furthermore, patients with severe psoriasis were less likely to receive beta-blockers, statins and platelet inhibitors after PCI.

Psoriasis and atherosclerosis are chronic inflammatory diseases characterized by T-helper (Th) 1-/Th17-driven inflammation and there is an apparent overlap of inflammatory mechanisms in these diseases [1,2]. Psoriasis has previously been associated with increased prevalence of conventional cardiovascular risk factors as well as cardiovascular morbidity and mortality [4-9,13-16]. Inflammation has also been suggested to contribute to post-PCI complications including restenosis, in-stent thrombosis and mortality [17]. Very limited data are available, however, on the impact of inflammatory diseases on the prognosis after PCI. A small study of patients with systemic lupus erythematosus demonstrated a worse 1-year outcome compared to controls while a very small study of patients with rheumatoid arthritis found no differences between groups [18,19]. On the other hand, the current demonstration of impaired prognosis after PCI in patients with severe psoriasis is likely to contribute to the adverse contemporary post-myocardial infarction prognosis found in patients with this disease, as well as in post-myocardial infarction subjects with systemic lupus erythematosus and rheumatoid arthritis [12,20-22].

In the present study, patients with psoriasis were more likely to receive statins, angiotensin converting enzyme (ACE) inhibitors/angiotensin 2 receptor antagonists, glucose-lowering drugs, and platelet inhibitors at baseline before index PCI than patients without psoriasis, supportive of previous reports of an increased prevalence of hypertension, hypercholesterolemia and diabetes mellitus in patients with psoriasis [7,23-29]. Also, in patients with psoriasis there was a higher proportion of NSAID users and medically managed depression compared to patients without psoriasis, both of which have been associated with impaired prognosis in patients with coronary artery disease [30-32]. After adjustments for these and other measured confounders, however, we found that severe psoriasis was a risk factor for adverse prognosis following first-time PCI, this suggests that the poor prognosis observed was not mainly driven by these differences in comorbidity. It is noteworthy that the proportion of high risk patients, i.e., where the index PCI was performed in the setting of MI, and that the rates of (index) primary PCI procedures (indicative of ST elevation myocardial infarction) were similar between patients with severe psoriasis and patients without psoriasis. Also, the proportion of patients treated with loop diuretics was similar irrespective of psoriasis status. This indicates a comparable distribution of severe congestive heart failure and is an important observation since we were unable to control for baseline and post-PCI left ventricular ejection fraction. Repeat revascularization rates after index PCI were also not statistically different between patients with and without psoriasis, but our study suggested differences in post-PCI pharmacological treatment, with patients with severe psoriasis receiving less beta-blockers, statins, and platelet inhibitors (Table 2). Although the reasons underlying the latter observation were not examined, significant chronic comorbidity has been associated with suboptimal post-myocardial infarction care and this particular finding may be an example of the treatment-risk paradox in coronary artery disease, i.e., that patients at increased risk receive relatively less evidence-based treatment [33]. In contrast, our previous report on prognosis after myocardial infarction in patients with psoriasis in the time period 2002–06 demonstrated no significant differences in post-myocardial infarction medical treatment, but that study included fewer patients and did not specifically evaluate the impact of psoriasis severity. Importantly, the analyses that controlled for these differences in post-PCI treatment gave results comparable to the primary analysis indicating that the impaired post-PCI prognosis in patients with severe psoriasis was not mainly driven by these differences. Taken together, the current finding of impaired post-PCI prognosis in patients with severe psoriasis therefore supports the notion that psoriasis-driven chronic inflammation per se may be an important determinant of the observed differences in post-PCI prognosis. This interpretation of the data reflects the sum of other current evidence on mechanisms underlying increased risk of cardiovascular disease in patients with chronic inflammatory diseases [3,6-11,14,15]. Accepted risk scores for prognosis after myocardial infarction may therefore underestimate the risk in these patients, as is the case for primary prevention, where traditional risk factor algorithms for coronary artery disease, e.g., the Framingham risk score, are unlikely to capture the full extent of increased risk [13,34-36].

Important strengths of the present study included the large number of participants, the ability to evaluate a psoriasis activity dose–response effect, the nationwide coverage in prospectively recorded registries, the contemporary clinical setting, and the completeness of follow-up. Furthermore, the use of nationwide registries of hospitalization data and dispensed prescriptions from all pharmacies in Denmark where healthcare is readily accessible and essentially free of charge minimized selection bias related to factors such as gender, age, socioeconomic status, healthcare insurance provision and job situation. Some limitations of our study should also be acknowledged. The results relied on the accuracy of the underlying registration in the registers, e.g., in terms of the study population and outcome measures. The register coding of revascularization procedures has not been validated but it is highly unlikely that the coding of these expensive and very specific treatments are erroneous. Also, misclassification in relation to psoriasis status is likely to be non-differential. The examined psoriasis cohort was based on prescriptions filled for topical vitamin D derivatives and our results may not apply to patients treated with other first-line antipsoriatic drugs, e.g., topical corticosteroids. However, there is no obvious reason to suspect important differences in baseline characteristics between patients treated with topical vitamin D or other first-line antipsoriatic drugs. Importantly, we have previously demonstrated that the criteria for the psoriasis diagnosis and severity classification in our registries were valid [14]. Because the Danish population is predominantly of Caucasian descent, the results should only be extrapolated to other ethnicities with caution. Finally, we were unable to account for some important cardiovascular risk factors, e.g., family history, obesity, and smoking, and other prognostic variables including coronary pathologic findings, PCI procedural variables, and left ventricular ejection fraction, and we cannot exclude the possibility unmeasured and/or residual confounding contributed to our findings. However, information on strong predictors of post-PCI adverse events, i.e., hypertension, diabetes mellitus, renal failure, and adherence to dual anti-platelet therapy were included [37].

## Conclusion

This first study of the post-PCI prognosis in patients with psoriasis demonstrated an increased risk of mortality and adverse cardiovascular events in patients with severe psoriasis compared to patients without psoriasis. These findings emphasize the increased burden of cardiovascular disease in patients with psoriasis and they call for further studies in this area of research.

## Methods

The study was a retrospective longitudinal cohort study using Danish nationwide prospectively recorded registries. The study complied with the Strengthening the Reporting of Observational Studies in Epidemiology (STROBE) recommendations [38].

### Ethics

This study was approved by The Danish Data Protection Agency (ref. 2008-41-2685). Approval from an ethics committee is not required for administrative register studies in Denmark.

### Study population and data sources

The present study population comprised all subjects from the entire Danish population, who underwent a first-time PCI (Nordic classification of surgical procedures code KFNG) in the period 2002–2009. Primary PCI in patients with ST elevation myocardial infarction was identified by the procedural codes KFNG02A and KFNG05A. Patients were followed until December 31 2009, emigration, or to the occurrence of an endpoint. Patients with PCI prior to the start of the study period were excluded. All Danish citizens have a unique lifelong personal civil registration number that enables linkage of information on an individual level across nationwide prospectively recorded registries. All medications dispensed from pharmacies were obtained from the Danish Registry of Medicinal Product Statistics, in which all dispensed prescriptions since 1995 are registered. Morbidity was obtained from the Danish National Patient Register in which hospital admissions, procedures, and diagnoses have been recorded since 1978 with the International Classification of Diseases (ICD) codes. Deaths were identified from the Central Population Register, wherein all deaths are recorded within 2 weeks. Socioeconomic status was defined as average yearly income in a period of up to 5 years prior to inclusion and divided into quintiles.

### Psoriasis identification

Patients with mild psoriasis were identified according to prescriptions claimed prior to study inclusion (index PCI) for topical treatment used exclusively for psoriasis, i.e., topical vitamin‐D derivatives (ATC D05AX). Subjects with severe psoriasis were identified by hospitalizations for psoriasis and psoriatic arthritis (ICD-10 L40 and M07.0-M07.3), respectively, from 1994 to index PCI, as validated previously [9,14].

### Medical treatment and comorbidity

Medical treatment for cardiovascular diseases and cardiovascular risk factors, including hypertension, dyslipidemia, and diabetes mellitus were identified by prescriptions for platelet inhibitors (ATC code: B01AC), beta-blockers (C07), ACE inhibitors/angiotensin 2 receptor antagonists (C09), loop diuretics (C03C), spironolactone (C03D), statins (C10A), and glucose-lowering drugs (A10) claimed up to 6 months prior to index PCI. Use of NSAID (M01A) and anti-depressive medication (N06A) was also ascertained. Initiation and adherence to cardiovascular secondary prophylaxis was evaluated at 30, 180, and 360 days from the index PCI. Comorbidity was defined by hospitalizations up to 12 months prior to the index PCI according to the Ontario myocardial infarction mortality prediction rule as adapted to ICD 10 [39].

### Outcomes

The primary study endpoint was all-cause mortality and a secondary composite endpoint was death, recurrent myocardial infarction (ICD-10 I21-I22) and stroke (ICD-10 I60, I61, I63, and I64). Repeat revascularization rates after index PCI were also determined, including PCI and coronary artery bypass grafting. The myocardial infarction and stroke diagnoses in the Danish registries have previously been found valid [40,41].

### Statistical analysis

Baseline characteristics were summarized and presented as means with standard deviations (SD) and percentages. Differences in baseline characteristics were assessed by analyses of variance (ANOVA) and by chi-square test for categorical covariates. IRs of study endpoints were reported as events per 1000 patient-years. The HRs and corresponding CIs were estimated by Cox regression models controlling for confounding factors including calendar year, patient age, gender, baseline use of medication and comorbidity, and socioeconomic data. A sensitivity analysis with start of follow-up 30 days from the index PCI and inclusion of post-PCI medication in the regression model was also done in order to assess the impact of differences in post-PCI treatment between the groups. Age- and sex-adjusted models, and a fully adjusted model with inclusion of age, sex, medication, comorbidity, and socioeconomic status, were fitted. Model assumptions, including absence of interaction between model covariates and the proportional hazard assumption were tested and found to be valid. A two-sided p <0.05 was considered statistical significant. All statistical analyses were performed with the SAS statistical software version 9.2 (Sas Institute inc., Cary, NC, USA) and Stata software version 11 (Statacorp, College st., TX, USA).

## Abbreviations

PCI: Percutaneous coronary intervention; IR: Incidence rate; HR: Hazard ratio; CI: 95% Confidence interval; NSAID: Non steroid anti-inflammatory drugs; ACE: Angiotensin-converting enzyme inhibitor; ARB: Angiotensin 2 receptor blocker.

## Competing interests

The authors have no competing interests.

## Authors' contributions

OA, JL, GG, LS, CTP, and PRH perceived and designed the study. OA, JL, and PRH drafted the manuscript. OA, JL, GG, and CTP carried out the statistical analyses. OA, JL, GG, JBO, MC, LS, CTP, and PRH participated in interpretation of the data, critical revision of the manuscript, and decision to submit for publication. All authors read and approved the final manuscript.

## Pre-publication history

The pre-publication history for this paper can be accessed here:

http://www.biomedcentral.com/1471-2261/12/79/prepub
